# Acyl CoA Binding Proteins are Required for Cuticle Formation and Plant Responses to Microbes

**DOI:** 10.3389/fpls.2012.00224

**Published:** 2012-10-08

**Authors:** Ye Xia, Keshun Yu, Qing-ming Gao, Ella V. Wilson, Duroy Navarre, Pradeep Kachroo, Aardra Kachroo

**Affiliations:** ^1^Department of Plant Pathology, University of KentuckyLexington, KY, USA; ^2^U.S. Department of Agriculture, Agricultural Research Service, Washington State UniversityProsser, WA, USA

**Keywords:** cuticle, plant defense, acyl CoA binding proteins, systemic acquired resistance, fatty acids

## Abstract

Fatty acids (FA) and lipids are well known regulators of plant defense. Our previous studies have shown that components of prokaryotic (plastidal) FA biosynthesis pathway regulate various aspects of plant defense. Here, we investigated the defense related roles of the soluble acyl CoA binding proteins (ACBPs), which are thought to facilitate the intracellular transport of FA/lipids. We show that *ACBP3* and *4* are required for maintaining normal lipid levels and that *ACBP3* contributes to the lipid flux between the prokaryotic and eukaryotic pathways. We also show that loss of *ACBP3*, *4*, or *6* impair normal development of the cuticle and affect both basal and resistance protein-mediated defense against bacterial and fungal pathogens. Loss of *ACBP3*, *4*, or *6* also inhibits the induction of systemic acquired resistance (SAR) due to the plants inability to generate SAR inducing signal(s). Together, these data show that ACBP3, ACBP4, and ACBP6 are required for cuticle development as well as defense against microbial pathogens.

## Introduction

In plants, *de novo* synthesis of fatty acids (FA) occurs exclusively in the plastids and is initiated by acetyl CoA carboxylase, which converts acetyl CoA to malonyl-CoA. The malonyl group is transferred from CoA to acyl carrier protein (ACP) carrying a phosphopantetheine prosthetic group to which the growing FA chains are esterified. The malonyl-ACP enters into a series of reactions that eventually result in the formation of 16 and 18 carbon saturated FAs, palmitate (16:0), and stearate (18:0; Ohlrogge and Browse, 1995; Kachroo and Kachroo, 2009). The 18:0 FA is desaturated to oleic acid (18:1) by stearoyl-acyl carrier protein-desaturases (SACPD) and in *Arabidopsis* the major SACPD isoform is encoded by *SSI2* (Kachroo et al., 2001, 2003, 2004, 2007, 2008; Venugopal et al., 2009; Xia et al., 2009; Mandal et al., 2012). The 16:0 and 18:1 FAs either remain inside plastids and enter the prokaryotic glycerolipid synthesis pathway or are exported as CoA thioesters to endoplasmic reticulum (ER) where they participate in glycerolipid synthesis via the eukaryotic pathway. The eukaryotic pathway leads to the synthesis of phospholipids such as phosphatidylcholine (PC), phosphatidylethanolamine (PE), and phosphatidylinositol (PI). The ER and plastids undergo extensive exchange of lipid precursors, including that of diacylglycerol (DAG), which is synthesized at both locations and serves as a precursor for the major plastidal galactolipids, monogalactosyldiacylglycerol (MGDG), and digalactosyldiacylglycerol (DGDG). This exchange and trafficking of lipid precursors requires their transport across various cellular compartments and is likely to involve proteins that can transport lipid precursors or promote physical associations between membranes (Moreau et al., 1998). Acyl CoA binding proteins (ACBPs) comprise one such family of proteins that can transport FA/lipid precursors (Kragelund et al., 1993; Yurchenko et al., 2009; Yurchenko and Weselake, 2011). The *Arabidopsis* genome encodes six isoforms of ACBPs, which have been well characterized for their structure, localization, expression, and substrate specificities (Chye, 1998; Li and Chye, 2003; Chen et al., 2008; Xiao and Chye, 2009; reviewed in Yurchenko and Weselake, 2011). ACBP1 and 2 are ankyrin-repeat containing membrane proteins that localize to the plasma membrane, ER, and small intracellular vesicles (Li and Chye, 2003). ACBP3, 4, 5, and 6 are soluble proteins that are present either in the extracellular space (ACBP3), or the cytosol (Xiao et al., 2008). The extracellular localization of ACBP correlates with the presence of a cleavable N-terminal signal sequence. ACBP4 and 5 show ∼81% homology at the amino acid level and contain kelch motifs, which represent potential sites for protein–protein interactions. Consistent with this, ACBP4 interacts with the ethylene-responsive element binding protein (Li et al., 2008), a transcription factor expressed in response to biotic and abiotic stresses (Büttner and Singh, 1997; Li et al., 2008).

Plant response to biotic stress involves the complex interplay of pathways induced by various phytohormones. These pathways interact antagonistically, additively, or synergistically to orchestrate plant defense (Doares et al., 1995; van Wees et al., 2000; Kunkel and Brooks, 2002; Glazebrook et al., 2003; Robert-Seilaniantz et al., 2011). Several of these phytohormones, including salicylic acid (SA) play important roles in non-host (species level), race-specific (also termed effector triggered immunity, ETI), and basal [also termed pathogen associated molecular patterns (PAMP) triggered immunity, PTI] resistance (Kachroo and Kachroo, 2007). SA is also important for the induction of systemic acquired resistance (SAR), one of the well studied induced defense responses, which primes for resistance against secondary pathogens (Dong, 2001). SAR is accompanied by a local and systemic increase in endogenous SA and the concomitant upregulation of a large set of defense genes, including those which encode pathogenesis related (PR) proteins (Boller et al., 1983; Carr et al., 1987; Loon et al., 1987; Ward et al., 1991; Gaffney et al., 1993; Uknes et al., 1993). SAR involves the generation of a mobile signal in the primary infected leaves, which upon translocation to the distal tissues, activates defense responses resulting in broad-spectrum resistance. In cucumber, the production of the mobile signal takes places within 3–6 h of inoculation with avirulent bacterial pathogen in the primary leaves (Smith-Becker et al., 1998). Studies in cucumber and *Arabidopsis* have shown that the primary infected leaf must remain attached for at least 4 h post infection for immunity to be induced in the distal tissues (Rasmussen et al., 1991; Chanda et al., 2011). The proper induction of SAR is dependent on several factors, including SA (MeSA; Park et al., 2007), the diterpenoid, dehydroabietylamine (DA, Chaturvedi et al., 2012), the nine carbon (C9) dicarboxylic acid, azelaic acid (AA, Jung et al., 2009), auxin (Truman et al., 2010), and the phosphorylated sugar, glycerol-3-phosphate (G3P, Chanda et al., 2011; Mandal et al., 2011). JA has been suggested to participate in SAR (Truman et al., 2007) as well, although its precise role remains debatable (Chaturvedi et al., 2008; Attaran et al., 2009; Xia et al., 2010).

The successful induction of SAR also requires an intact cuticle, a hydrophobic layer that covers the aerial surfaces of the plant (Xia et al., 2009, 2010). The cuticle layer in *Arabidopsis* leaves is composed of cuticular waxes and cutin monomers and fatty acid (FA) flux plays an important role in their formation. The plastidal C16 and C18 FAs are exported outside plastids and extended to very long chain FAs (VCLF) in the ER compartment. The VLCFAs are converted into cuticular waxes either by deactivation of acyl-CoA thioesters to release FAs, by conversion of aliphatic esters via the condensation of an acyl moiety with a primary alcohol, or via reductive pathways that convert acyl-CoAs to primary alcohols or aldehydes (see review by Kachroo and Kachroo, 2009). Alkanes, which are the major components of cuticular wax, are generated from aldehydes and are subsequently converted to secondary alcohols and ketones. Cutin component of cuticle is formed by the polymerization of hydroxy group of C16 and C18 ω-hydroxy FAs with the carbonyl group of another monomer (Molina et al., 2006; Pollard et al., 2008). Cutin biosynthesis is also dependent on FA oxidases, acyl-activating enzymes, and acyltransferases. Detailed characterization of two cuticle defective mutants, *acp 4* and *glabrous* (*gl*) *1* has shown that the cuticle defect impairs the plant’s ability to respond to the mobile SAR signal but does not affect its ability to generate it (Xia et al., 2009, 2010). Consistent with this result, mechanical abrasion of cuticle of distal leaves compromised SAR in wild-type (wt) plants (Xia et al., 2009). The SAR defect in *acp4* plants is likely not associated with their reduced FA pool. This is because mutations in different membrane-localized FA desaturases (introduce double bonds in specific FAs of membrane lipids; e.g., FAD2, FAD3, FAD7, FAD8) reduce the levels of corresponding FAs but do not inhibit the induction of SAR (Xia et al., 2010). The precise contribution of cuticle in SAR mobile signal perception remains unknown.

The fact that plant cuticle comprises a complex mixture of VLCFA derivatives formed upon elongation of plastidal C16 and C18 FAs suggests that lipid/FA trafficking might play an important role in cuticle development. Based on this assumption, we evaluated the roles of ACBP3, ACBP4, and ACBP6 in cuticle development and thereby plant defense. We show that mutations in *ACBP3*, *ACBP4*, or A*CBP6* impair normal development of the cuticle to varying levels and affect both basal and race-specific defense against microbial pathogens. These *acbp* mutants are also defective in SAR. However, unlike *acp4* and *gl1* plants, the *acbp* mutants were competent in the perception of SAR signal but compromised in its generation. Our data suggest that ACBP3, ACBP4, and ACBP6 may be involved in the transport of FAs and/or lipid species required for the proper development of the plant cuticle as well as the generation of the mobile SAR signal.

## Results

### The *acbp*3 and *acbp*4 plants are affected in lipid metabolism

**Figure 1:**
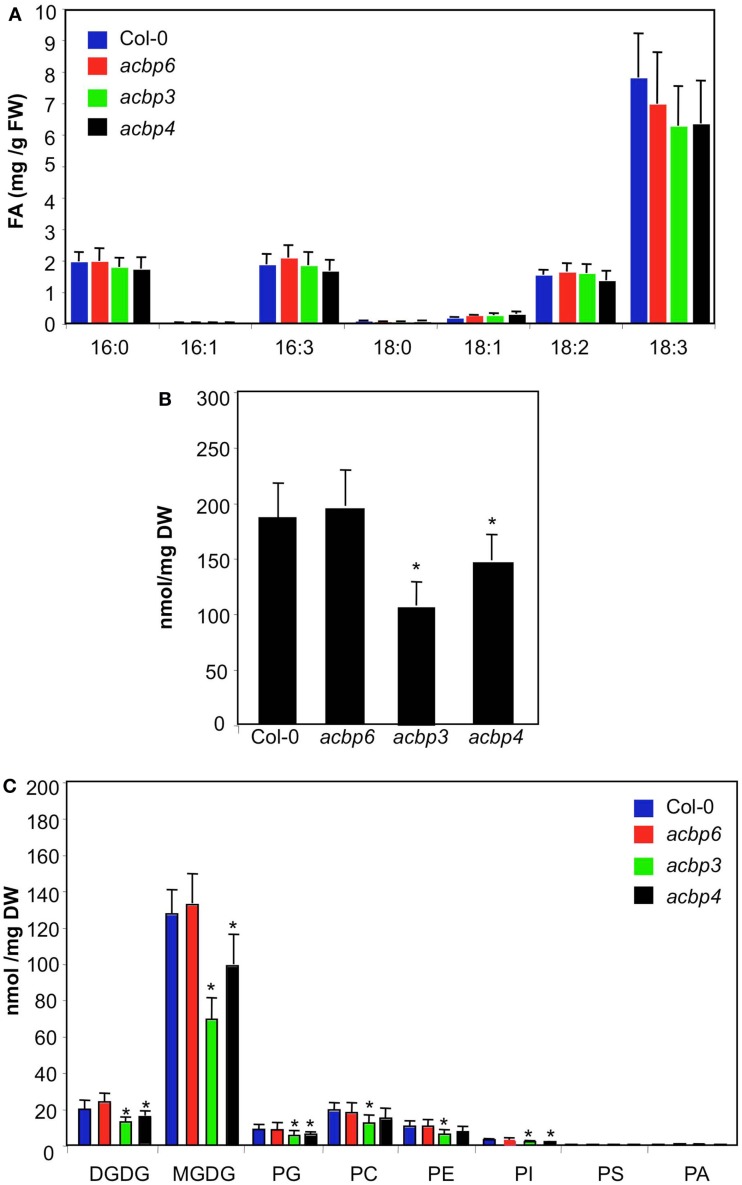
**FA and lipid levels in *ACBP* KO plants**. **(A)** Levels of total FAs in 4-week-old Col-0 and *acbp* mutant plants. The values are presented as mean of six to eight replicates. FW indicates fresh weight. The error bars represent SD. The experiment was repeated five times with similar results. **(B)** Total lipid levels in Col-0 and *acbp* mutant plants. The values are presented as a mean of five replicates. The error bars represent SD. Asterisks denote a significant difference with Col-0 (*t*-test, *P* < 0.05). DW indicates dry weight. **(C)** Profile of total lipids extracted from Col-0 and *acbp* mutant plants. The values are presented as a mean of five replicates. The error bars represent SD. Asterisks denote a significant difference with Col-0 (*t-*test, *P* < 0.05). Symbols for various components are: DGD, digalactosyldiacylglycerol; MGD, monogalactosyldiacylglycerol; PG, phosphatidylglycerol; PC, phosphatidylcholine; PE, phosphatidylethanolamine; PI, phosphatidylinositol; PS, phosphatidylserine; PA, phosphatidic acid.

**Figure A1:**
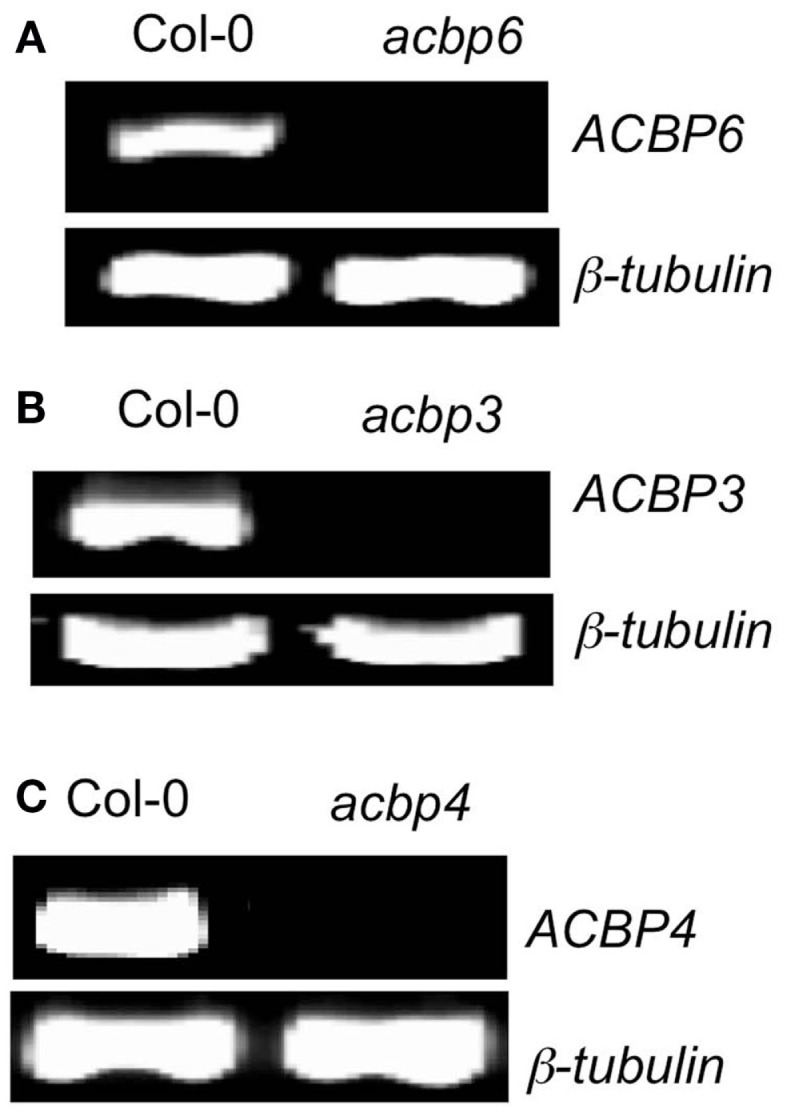
**Reverse transcription-PCR analysis showing *ACBP* transcript levels in wild-type (Col-0) and *acbp3* (A), *acbp4* (B), and *acbp6* (C) mutant plants**. The level of β*-tubulin* was used as an internal control to normalize the amount of cDNA template. The RT-PCR analysis was repeated with two independent cDNA templates per genotype.

**Figure A2:**
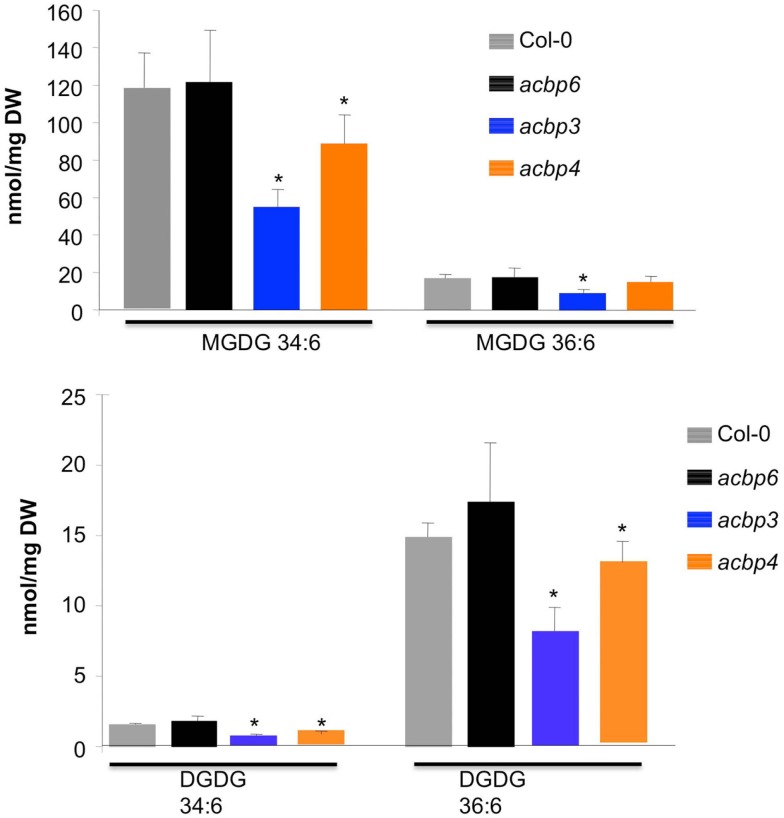
**Levels of MGDG and DGDG containing 34:6 or 36:6 FAs in Col-0 and *acbp* mutant plants**. The values are presented as a mean of five replicates. The error bars represent SD. Asterisks denote significant differences with Col-0 (*t-*test, *P* < 0.05).

We hypothesized that *ACBP3*, *ACBP4*, *ACBP5*, *ACBP6*, which encode soluble proteins, were likely to play a role in FA/lipid flux. The encoded proteins are predicted to localize to the cytoplasm (ACBP4, 5, and 6) or the extracellular space (ACBP3; Xiao and Chye, 2009). We attempted to isolate knock-out (KO) lines in each of these genes but were only able to isolate homozygous T-DNA insertions in *ACBP3* (At4g24230), *ACBP4* (At3g05420), and *ACBP6* (At1g31812) genes. Similar lines were used in previous studies where the KO mutations were confirmed by functional complementation with the respective wt gene (Chen et al., 2008; Xiao et al., 2008, 2010). The KO mutations were verified by RT-PCR, which confirmed the absence of detectable transcripts in the respective lines (Figure A1 in Appendix). All *acbp* mutant plants showed wt-like morphology (data not shown) and wt-like FA profiles (Figure 1A). The *ACBP* KO plants also showed wt-like levels of long chain FAs (data not shown). Interestingly, in contrast to their FA profiles, the *acbp3* and *acbp4* mutant plants showed significant reduction in their total lipid levels, whereas *acbp6* plants accumulated wt-like levels of total lipids (Figure 1B). Analysis of individual lipid levels showed reduced levels of MGDG, DGDG, PG, PC, PE, and PI in *acbp3* and reduced levels of MGDG, DGDG, PG, and PI in *acbp4* plants (Figure 1C). Analysis of FA species present on the plastidal lipids MGDG or DGDG lipids showed that *acbp3* and *acbp4* plants were reduced in lipid subspecies that were either made in plastids (contain 16:3 and 18:3 FAs) or imported from outside (both FA species are C18; Figure A2 in Appendix). Together, these results indicate that *ACBP3* and *4* contribute to membrane lipid synthesis and the lipid flux between the prokaryotic and eukaryotic pathways.

### The *acbp* plants are defective in their cuticle

**Figure 2:**
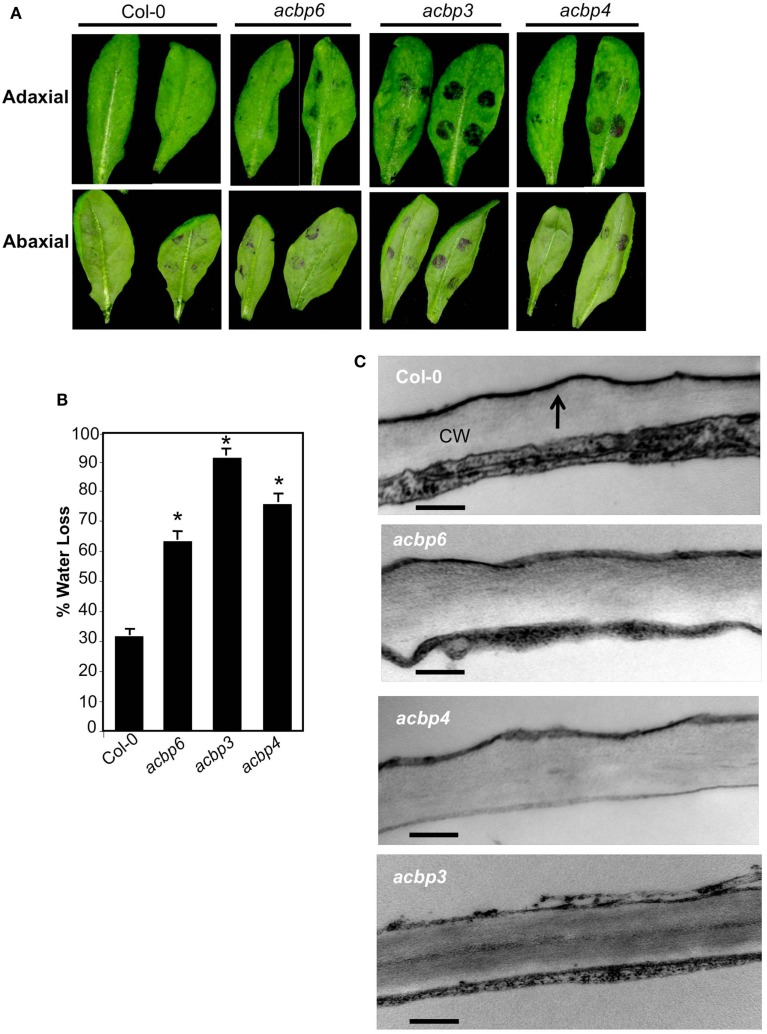

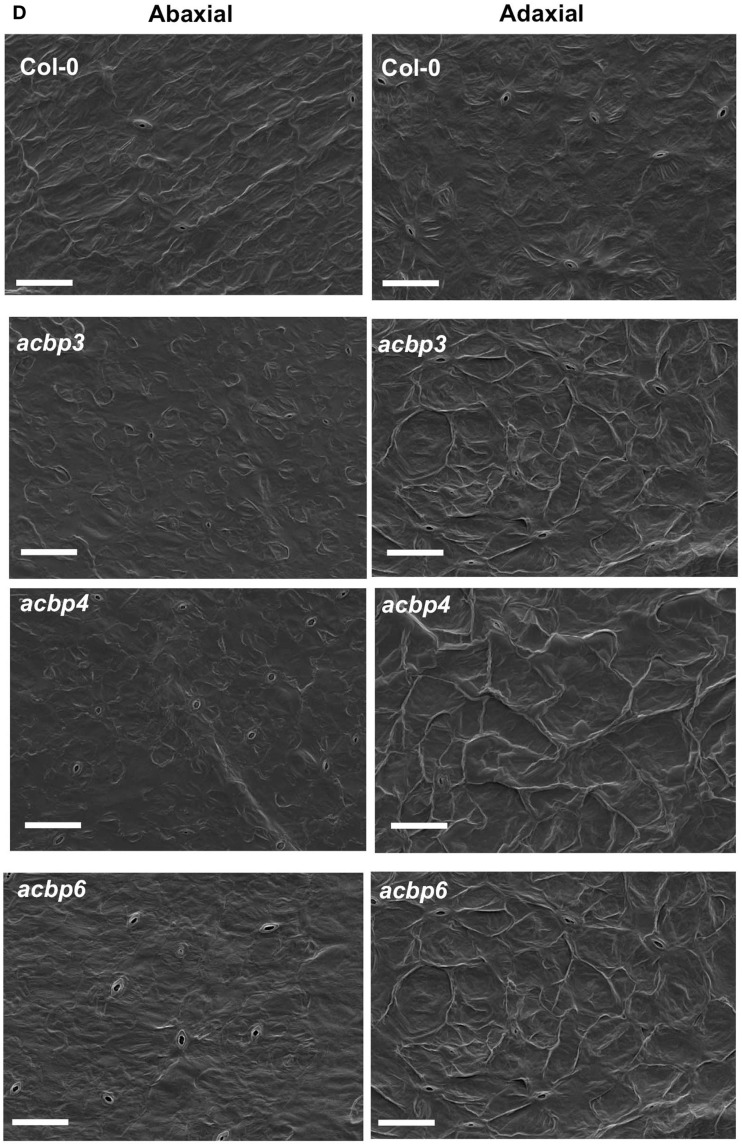
**Evaluation of cuticle associated phenotypes in *acbp* mutant plants**. **(A)** Toluidine blue stained leaves from 4-week-old plants. The stain was spotted on the adaxial or abaxial surface of the leaves and the leaves were washed with water after 20 (left) or 40 min (right) incubations. The experiment was repeated three times with similar results. **(B)** Measurement of water lost from the leaves subjected to drought conditions for 4 days. The error bars represent SD (*n* = 25). Asterisks denote a significant difference with Col-0 (*t-*test, *P* < 0.05). The experiment was repeated three times with similar results. **(C)** Transmission electron micrographs showing cuticle layer on adaxial surface of leaves from indicated genotypes. At least two independent leaves were sectioned and analyzed. Arrows indicate cuticle. CW indicated cell wall (scale bars, 50 nm). **(D)** Scanning electron micrographs showing adaxial (left panel) and abaxial (right panel) surface of leaves from indicated genotypes (scale bars, 200 μM). Two-three independent leaves were analyzed per genotype.

**Figure A3:**
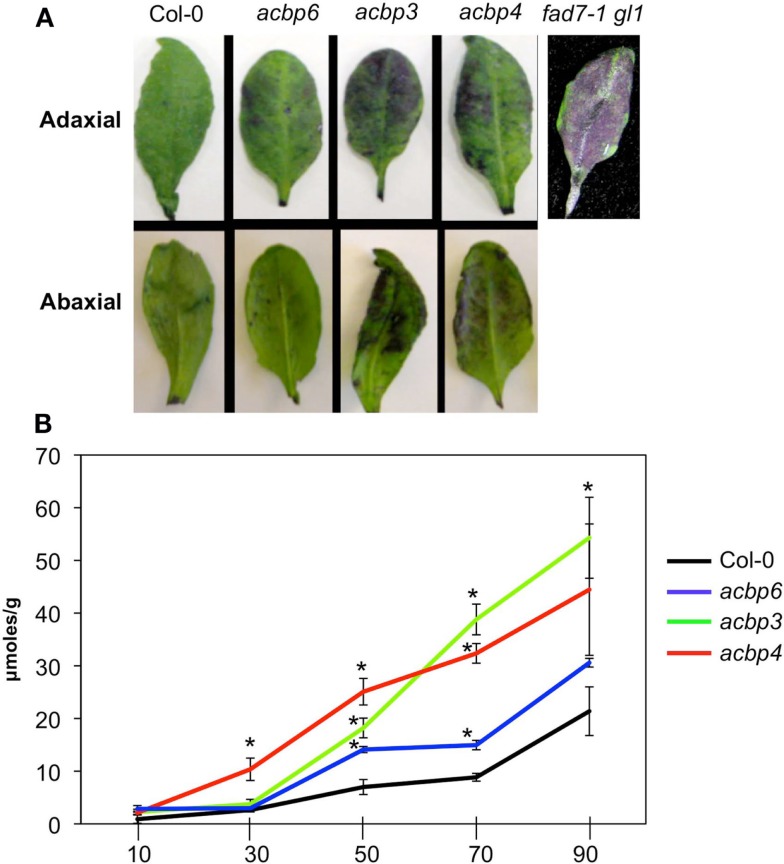
**Cuticular phenotypes of *acbp* plants**. **(A)** Whole leaf toluidine blue staining. Leaves were incubated in the stain for 10 min and the experiment was repeated four times with similar results. **(B)** A time-course measurement of chlorophyll leaching in various genotypes at indicated times. The values are presented as a mean of four replicates. The error bars represent SD (*P* < 0.05). The experiment was repeated four times.

To test if the altered lipid levels in *acbp3* and *acbp4* plants impaired cuticle formation, we analyzed the cuticular phenotypes of these plants. We first stained wt and *acbp* leaves with toluidine blue, a hydrophilic dye that only penetrates leaves with permeable cuticles (Tanaka et al., 2004). Toluidine blue penetrated *acbp3* and *acbp4* leaves, staining these blue, suggesting cuticular permeability (Figure 2A; Figure A3A in Appendix). Interestingly, toluidine blue also stained *acbp6* leaves, although the staining was less intense. The adaxial surface of all *acbp* mutant plants stained more compared to the abaxial surfaces (Figure 2A; Figure A3A in Appendix). Moreover, *acbp* mutants showed considerably less staining compared to *fad7-1 gl1* leaves (Figure A3A in Appendix), suggesting that the cuticular defects of *acbp* mutants were likely less pronounced than that of the *fad7-1 gl1* plants. Increased permeability to toluidine blue correlated with water lost from the leaves when subjected to drought stress (Figure 2B); consistent with increased toluidine blue staining, the *acbp3* plants lost more water followed by *acbp4* and *acbp6* plants. Similarly, *acbp3* plant showed highest leaching of chlorophyll followed by *acbp4* and *acbp6* (Figure A3B in Appendix).

To confirm that the cuticle is indeed defective in *acbp* leaves, we analyzed the outermost cell wall of the epidermis by transmission electron microscopy (TEM). As expected, the cuticle of wt leaves appeared as a continuous and regular electron-dense osmophilic layer outside the cell wall (Figure 2C, marked by an arrow). In comparison, the cuticle of *acbp4* and *acbp6* mutants showed both electron-dense and -opaque regions. Strikingly, the cuticle of *acbp3* plants was thin, highly irregular, and electron-opaque. Scanning electron micrograph (SEM) analysis of wt and *acbp* leaf surfaces showed increased folding on the adaxial surface (Figure 2D, left panels). In comparison, their abaxial surfaces did not exhibit obvious alterations (Figure 2D, right panels).

**Figure 3:**
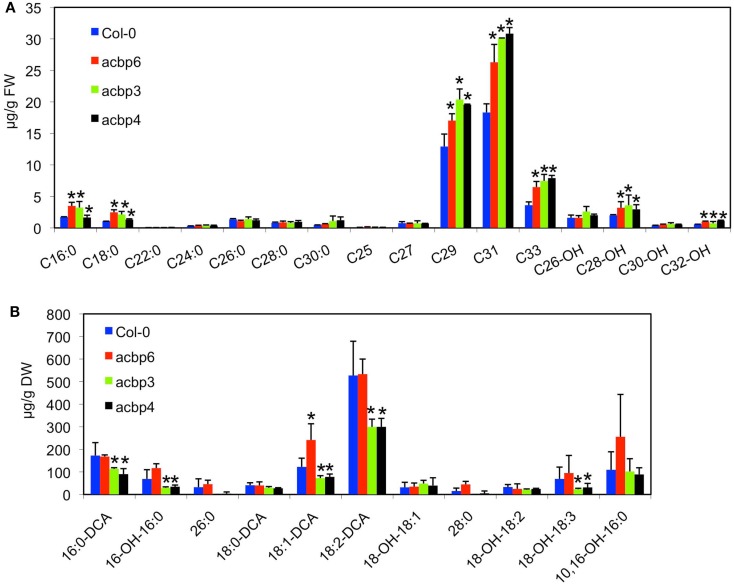
**Biochemical profiles of cuticular wax and cutin monomers in *acbp* mutant plants**. **(A)** Analysis of wax components from leaves of 4-week-old Col-0 and *acbp* plants. C16:0-C30:0 are FAs, C25-C33 are alkanes, C26-OH-C32-OH are primary alcohols. The values are presented as a mean of five replicates. The error bars represent SD. Asterisks denote a significant difference with Col-0 (*t-*test, *P* < 0.05). DW indicates dry weight. **(B)** Analysis of lipid polyester monomer content of 4-week-old Col-0 and *acbp* plants. Error bars in **(A,B)** represent SD. Statistical significance was calculated using Student’s *t*-test (*t-*test, *P* < 0.05). Symbols for various components are: 16:0-DCA, 1,16-hexadecane dioic acid; 16-OH-16:0, 16-hydroxyhexadecanoic acid; 10,16-OH-16:0, 10,16-dihydroxyhexadecanoic acid; 18:0-DCA, 1,18-octadecane dioic acid; 18:1-DCA, 1,18-octadecene dioic acid; 18-OH-18:1, 18-hydroxyoctadecenoic acid, 18:2-DCA, 1,18-octadecadiene dioic acid; 18-OH-18:2, 18-hydroxyoctadecadienoic acid; 18-OH-18:3, 18-hydroxyoctadecadienoic acid.

To determine if this defect in cuticle structure was associated with alterations in the content and/or composition of cuticular waxes or cutin polyester monomers, we compared levels of waxes and cutin monomers of wt and *acbp* leaves. Notably, all *acbp* mutants showed significant increases in FA (16:0, 18:0), alkanes (C29, C31, and C33), and primary alcohols (C28-OH, C32-OH) compared to wt plants (Figure 3A). In contrast to cuticular wax, *acbp3* and *4* plants showed greatly reduced levels of cutin monomers (Figure 3B). The decrease was more pronounced in three major monomers, 16:0-, 18:1-, and 18:2-dicarboxylic acids (DCA). Although, the *acbp6* plants showed nominal increase in 18:1-DCA, the levels of most other cutin monomers were similar to that of wt plants. Increased biosynthesis of cuticular components has also been observed in several *Arabidopsis* mutants that show abnormal cuticle (Schnurr et al., 2004; Kurdyukov et al., 2006; Bessire et al., 2007; Voisin et al., 2009). Together, these data suggest that loss of *ACBP3*, *4*, and *6* leads to varying levels of cuticular defects.

### The *acbp* plants show compromised SAR and resistance to fungal and bacterial pathogens

**Figure 4:**
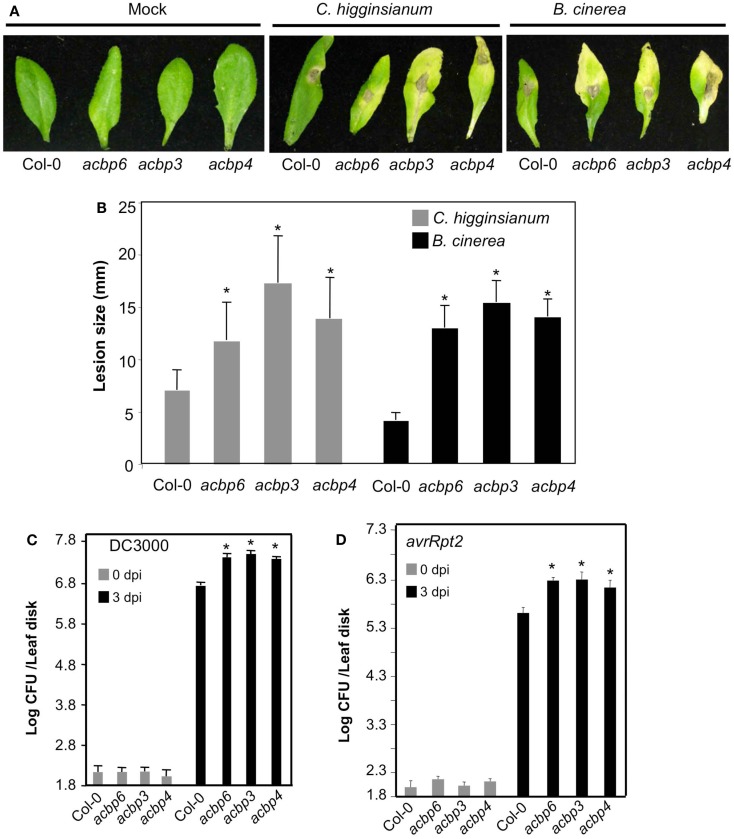
**The *acbp* mutant plants show compromised response to fungal and bacterial pathogens**. **(A)** Disease symptoms on indicated genotypes spot-inoculated with water or 10^6^ spores/ml of *C. higginsianum* or *B. cinerea*. The experiment was carried out five times and three of these showed enhanced susceptibility in *acbp* plants. **(B)** Lesion size in spot-inoculated genotypes. The plants were spot-inoculated with 10^6^ spores/ml of *C. higginsianum* and the lesion size was measured from 20 to 30 independent leaves at 6 dpi. Statistical significance was determined using Student’s *t*-test. Asterisks indicate data statistically significant from that of control (Col-0; *P* < 0.05). Error bars indicate SD. **(C)** Growth of virulent *P. syringae* on leaves from Col-0 or *acbp* mutant plants. Error bars indicate SD. Asterisks indicate data statistically significant from that of control (Col-0; *P* < 0.05, *n* = 4). **(D)** Growth of avirulent (*avrRpt2*) *P. syringae* strains on Col-0 or *acbp* mutant plants. Error bars indicate SD. Asterisks indicate data statistically significant from that of control (Col-0; *P* < 0.05, *n* = 4). Bacterial growth presented as the LOG of colony forming units (CFU) per leaf disk, was monitored at 0 and 3 dpi. Experiments in **(C,D)** were repeated six times each with similar results.

**Figure A4:**
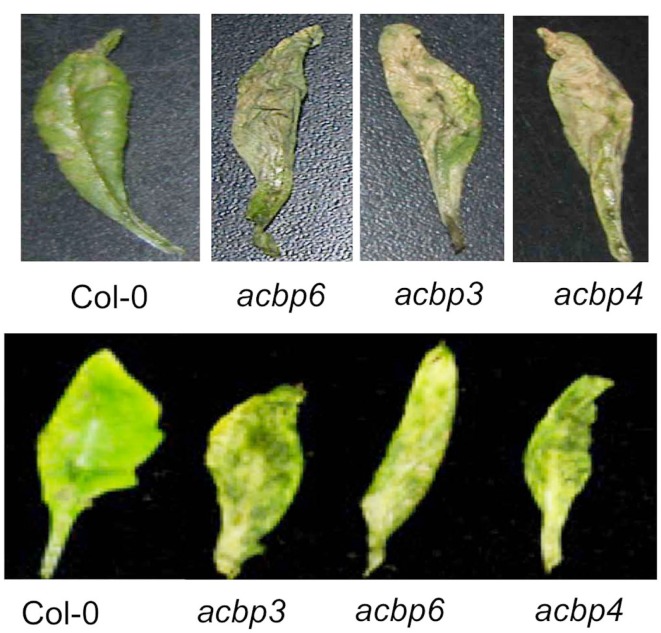
**Disease symptoms on indicated genotypes spray-inoculated with 10^6^ spores/ml of *C. higginsianum***. The experiment was carried out five times, three of which showed enhanced susceptibility in *acbp* plants, while the remaining two showed wild-like infection phenotypes on *acbp* plants. The upper and lower panels show two independent experiments that showed enhanced susceptibility in *acbp* plants.

Since cuticle plays an important role in defense against fungal pathogens, we next evaluated the response of *acbp3*, *acbp4*, *acbp6* mutants to the necrotrophic pathogen *Botrytis cinerea* and a hemibiotrophic fungal pathogen *Colletotrichum higginsianum*. Interestingly, in the majority of experiments, *acbp* mutants showed enhanced susceptibility to *B. cinerea* and *C. higginsianum*; spray and spot inoculations showed significantly larger lesions on *acbp* leaves (Figures 4A,B; Figure A4 in Appendix). However, in two of five experiments no noticeable difference in infection symptoms was observed between Col-0 and *acbp* mutants (see Figure legends for detail). In comparison, all *acbp* plants consistently showed enhanced susceptibility to virulent (DC3000) and avirulent (*avrRpt2*) strain of the bacterial pathogen *Pseudomonas syringae* (Figures 4C,D). Together, these data suggested that loss of *ACBP6*, *ACBP3*, and *ACBP4* impaired basal and race-specific defense against fungal and bacterial pathogens.

**Figure 5:**
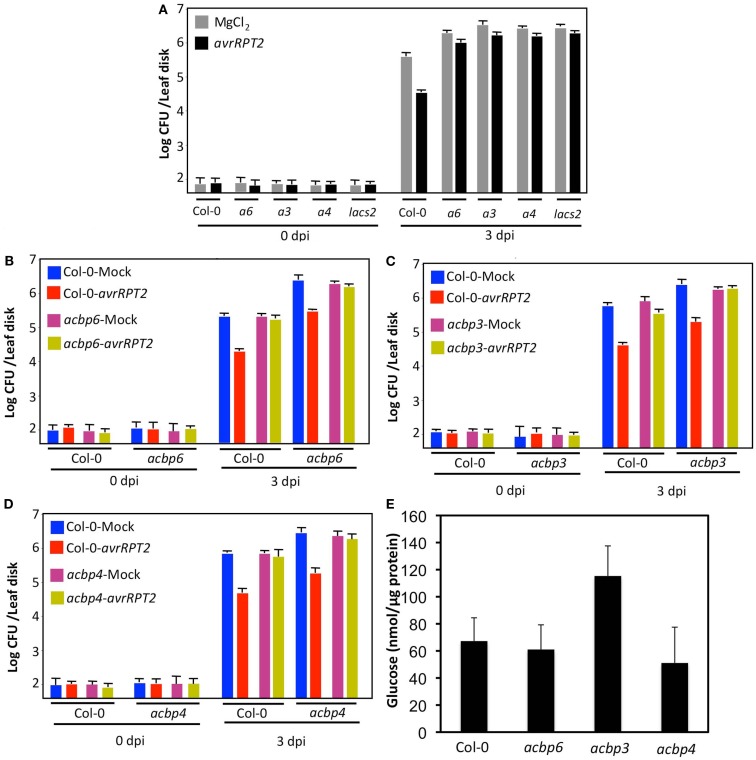
**The *acbp* mutants show compromised SAR**. **(A)** SAR response in Col-0 and *acbp6* (*a6*), *acbp3* (*a3*), *acbp4* (*a4*), and *lacs2* plants. Primary leaves were inoculated with MgCl_2_ (gray bars) or *P. syringae* containing *avrRpt2* (black bars). The distal leaves were inoculated with the virulent *P. syringae* and growth of the virulent bacteria was monitored at 3 dpi. The SAR impaired *lacs2* plants were used as a positive control (Xia et al., 2009). This experiment was repeated six times with similar results. Asterisk denotes significant difference from plants of the same genotype pre-infiltrated with MgCl_2_ (*t*-test, *n* = 4, *P* < 0.0001). **(B–D)** SAR response in Col-0 and *acbp* plants infiltrated with exudates (Ex) collected from wt or *acbp* plants that were treated either with MgCl_2_ (blue and pink bars) or *P. syringae* expressing *avrRpt2* (red and yellow bars). Error bars indicate SD (*n* = 4). Statistical significance was calculated using Student’s *t*-test (*P* < 0.0001). Experiments shown in **(B–D)** were repeated twice with similar results. Bacterial growth presented as the LOG of colony forming units (CFU) per leaf disk, was monitored at 0 and 3 dpi. **(E)** Glucose levels in petiole exudates collected from indicated genotypes. Error bars indicate SD. The experiment was repeated twice and the second repeat showed wild-type-like glucose levels in *acbp3* plants. Error bars indicate SD (*n* = 3). No statistical significance was observed in the levels from different genotypes per Student’s *t*-test.

Previously, we showed that intact cuticle is required for the normal induction of systemic immunity in plants (Xia et al., 2009, 2010). To determine if the cuticle defect in *acbp* plants affected systemic immunity, we next tested their abilities to induce SAR. The plants were first infiltrated with MgCl_2_ or an avirulent strain of *P. syringae* (*avrRpt2*), 48 h later distal leaves of both sets of plants were challenged with a virulent strain of *P. syringae* (DC3000). The proliferation of virulent bacteria was monitored at 0 and 3 dpi. The wt plants previously inoculated with *avrRpt2* *P. syringae*, showed ∼10-fold reduced growth (*P* < 0.0001) of virulent bacteria compared to plants previously infiltrated with MgCl_2_ (Figure 5A). In contrast, the *acbp* plants showed only ∼1- to 1.5-fold reduction in the growth of virulent bacteria at 3 dpi (these differences were not statistically significant), when pre-exposed to *avrRpt2* bacteria. Thus, all *acbp* mutant plants were defective in their ability to induce SAR.

The cuticular defect in *acp4* plants impairs their ability to perceive the SAR signal but not their ability to generate the mobile SAR signal. To test if this were also the case with the *acbp* mutants, we evaluated the response of wt and *acbp* plants to petiole exudates collected from pathogen infected leaves of wt and *acbp* mutant plants. The wt or *acbp* leaves were infiltrated with MgCl_2_ or *avrRpt2* bacteria and petiole exudates collected from these leaves were injected into the leaves of a fresh set of wt and the corresponding *acbp* mutant plants. Distal leaves of the exudate-infiltrated plants were then inoculated with virulent bacteria and proliferation of virulent bacteria monitored at 0 and 3 dpi (Figures 5B–D). As expected, exudates from *avrRpt2*-infected wt plants conferred protection against virulent pathogen in wt plants (*P* < 0.0001). The exudates from wt plants also conferred protection against virulent pathogen in *acbp* plants (*P* < 0.0001), suggesting that *acbp* plants were able to perceive the SAR signal. In contrast to wt, exudates from *avrRpt2*-infected *acbp* plants were unable to confer SAR in wt or respective *acbp* plants, suggesting that *acbp* plants are defective in generating the mobile SAR signal. To determine if this was due to defective exudation, we monitored glucose levels in petiole exudates collected from wt and *acbp* plants. Petiole exudates collected from untreated plants showed wt-like levels of glucose in *acbp* plants (Figure 5E), suggesting that *acbp* plants are not defective in the exudation process. A higher level of glucose seen in *acbp3* petiole exudates was only seen in one of two experiments, and was not statistically significant. Together, these results suggest that *acbp* plants are defective in the generation of SAR signal but competent in its perception. Interestingly, this phenotype is just the reverse of that observed in other cuticle defective *acp4* and *gl1* plants, which are defective in perception of the SAR signal (Xia et al., 2009, 2010).

**Figure 6:**
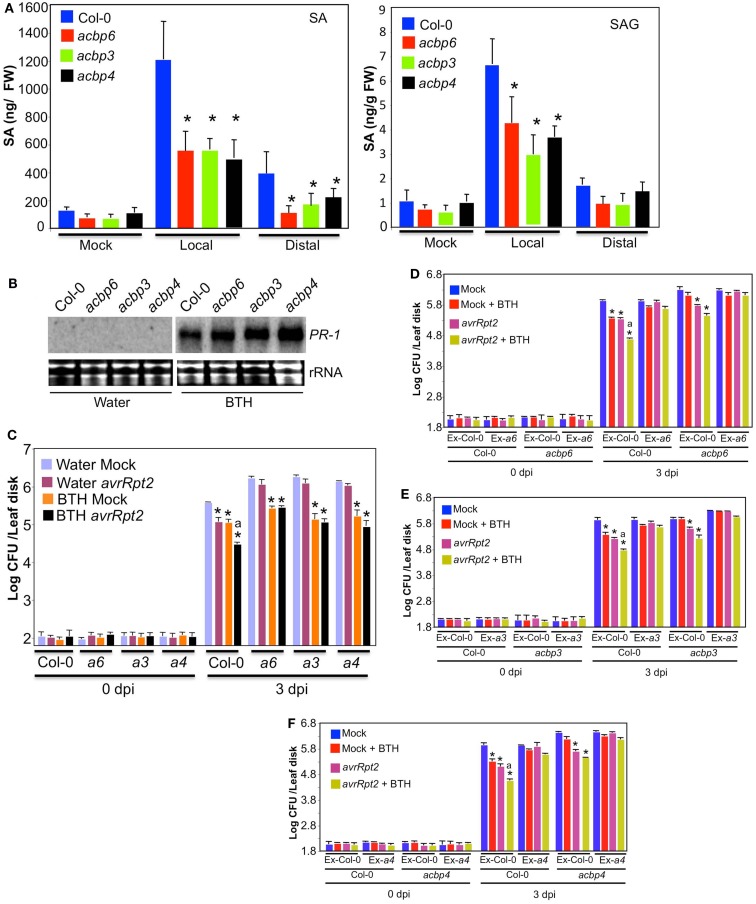
**The *acbp* mutants accumulate reduced levels of SA**. **(A)** SA and SAG levels in local (inoculated) and distal (uninoculated) leaves of Col-0 and *acbp* plants inoculated with MgCl_2_ or *P. syringae* expressing *avrRpt2*. Leaves were harvested at 3 dpi. Error bars indicate SD. Asterisks indicate data statistically significant from that of control (Col-0; *P* < 0.05, *n* = 4). The experiment was repeated twice with similar results. **(B)** RNA gel blot showing transcript levels of *PR-1* gene in plants treated with water or BTH for 48 h. Ethidium bromide staining of total RNA was used as the loading control. The experiment was repeated twice with similar results. **(C)** SAR response in Col-0 and *acbp* plants pretreated with water (purple and pink bars) or the SA analog BTH (orange and black bars) for 48 h prior to mock (purple and orange bars) or *avr* (pink and black bars) inoculation. The error bars represent SD (*n* = 4). Asterisks denote statistical differences from water and mock treated plants of corresponding genotype (*t*-test *P* < 0.001). Statistical difference from BTH and mock treated plants is indicated by “a” (*P* < 0.001). The experiment was repeated three times with similar results. **(D–F)** SAR response in Col-0 and *acbp* plants infiltrated with exudates (Ex) collected from wt or *acbp* plants that were treated either with MgCl_2_ (mock, blue, and red bars) or *P. syringae* expressing *avrRpt2* (pink and yellow bars). Exudates were mixed with water (blue and pink bars) or 100 μM BTH (red and yellow bars) prior to infiltration into a fresh set of plants. Error bars indicate SD (*n* = 4). Statistical significance was calculated using Student’s *t*-test. Asterisks denote statistical differences from mock + water-treated plants (blue bars) of corresponding genotype (*t*-test *P* < 0.005). Statistical difference from *avrRpt2* + water-treated plants (pink bars) is indicated by “a” (*P* < 0.01). Bacterial growth presented as the LOG of colony forming units (CFU) per leaf disk, was monitored at 0 and 3 dpi. Experiments in **(B–D)** were repeated twice with similar results. *a3*, *a4*, *a6* indicate *acbp3*, *acbp4*, and *acbp6*, respectively.

**Figure A5:**
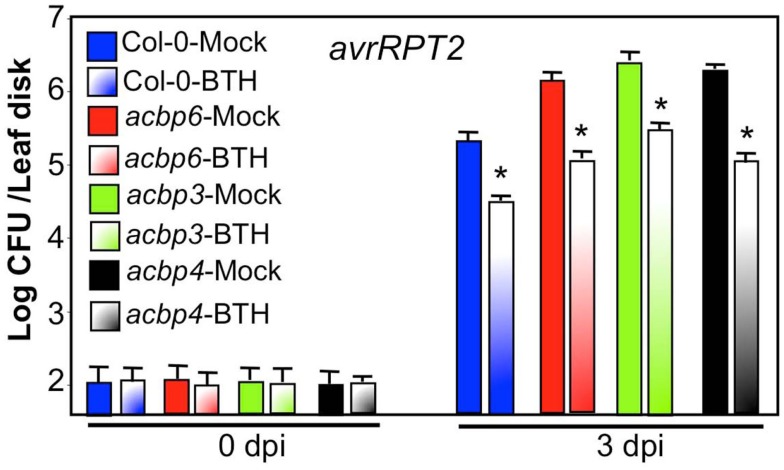
**Pathogen response of Col-0 and *acbp* plants after exogenous application of the SA analog BTH**. The Col-0 and *acbp* plants were pretreated with water (mock, solid bars) or 100 μM BTH (shaded bars) prior to infiltration with *P. syringae* expressing *avrRpt2*. Bacterial growth presented as the LOG of colony forming units (CFU) per leaf disk, was monitored at 0 and 3 dpi. Error bars indicate SD (*n* = 4). Statistical significance was calculated using Student’s *t*-test (*P* < 0.001). The experiment was repeated twice with similar results.

**Figure A6:**
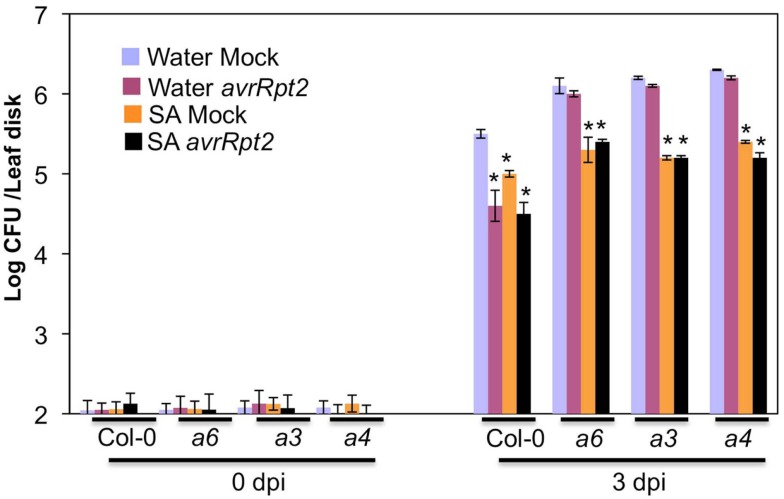
**Systemic acquired resistance response in Col-0 and *acbp6* (*a6*), *acbp3* (*a3*), *acbp4* (*a4*) plants, pretreated with water (blue and pink bars) or 50 μM SA (orange and black bars) prior to infiltration with MgCl_2_ (mock, blue, and orange bars) or *P. syringae* expressing *avrRpt2* (pink and black bars)**. Bacterial growth presented as the LOG of colony forming units (CFU) per leaf disk, was monitored at 0 and 3 dpi. Error bars indicate SD (*n* = 4). Asterisks denote statistical differences from water + mock-inoculated plants (blue bars) of corresponding genotype, calculated using Student’s *t*-test (*P* < 0.001).

Since SA plays a critical role in basal, R-mediated resistance, and SAR, we next tested if the *acbp* mutant plants were competent in pathogen responsive accumulation of SA. SA levels in wt and *acbp* plants were determined before and after inoculation of *P. syringae* expressing *avrRpt2*. As expected, wt plants inoculated with avirulent pathogen showed a significant increase in both free SA and SA glucoside (SAG) in their primary (inoculated) as well as distal uninoculated tissues. Although the *acbp* plants also showed an increase in SA and SAG levels in the primary tissues, levels of SA/SAG in these were significantly lower compared to wt plants (Figure 6A). Thus, impaired SAR in *acbp* plants correlated with their inability to accumulate SA. The *acbp* mutants were responsive to SA or its biologically active analog BTH [benzo (1,2,3) thiadiazole-7-carbothioic acid] and induced wt-like expression of the marker gene *PR-1* (Figure 6B, data shown for BTH treatment). This suggested that the *acbp* mutants were sensitive to exogenous SA and the compromised local defenses and SAR in *acbp* mutants was not related to perception of SA. We next assayed the effect of exogenously supplied BTH on basal- and R-mediated resistance and SAR. The wt and *acbp* plants were treated with BTH for 48 h prior to mock or pathogen inoculations. Exogenous whole plant BTH application increased local resistance against both virulent and avirulent pathogens in wt and *acbp* mutants (Figure A5 in Appendix, *P* < 0.001, data not shown for virulent pathogen). In contrast, and unlike wt plants, BTH pretreated mock- and *avrRpt2* inoculated plants supported similar growth of virulent bacteria, suggesting that BTH treatment was unable to confer SAR in *acbp* plants even though it did improve resistance compared to water-treated plants (Figure 6C). To test this further, we collected petiole exudates from wt and *acbp* leaves that were infiltrated with MgCl_2_ (mock) or *avrRpt2* bacteria and mixed these with water, BTH, or SA prior to infiltrating these into the primary leaves of a fresh set of wt and *acbp* plants. The distal leaves of this second set of plants were then inoculated with virulent bacteria and proliferation of the virulent bacteria monitored at 0 and 3 dpi (Figure 6D–F, see Figure A6 in Appendix for SA related data). As expected, exudates from *avrRpt2*-infected wt plants conferred protection against virulent pathogen in wt plants (compare pink and blue bars in Ex-Col-0 treatment for each genotype *P* < 0.005). Similarly, exudates from *avrRpt2*-infected wt plants also conferred protection against virulent pathogen in *acbp3*, *acbp4*, and *acbp6* plants, thus confirming their inability to generate SAR signal (Figures 5B–D, *P* < 0.0001, compare blue and red bars for each genotype). In contrast, petiole exudates from *avrRpt2*-infected *acbp3*, *acbp4*, or *acbp6* plants were unable to confer resistance against virulent pathogen in wt plants or themselves (Figures 6D–F, compare pink and blue bars in Ex-*a3*/*a4*/*a6* treatment for each genotype, also see Figures 5B–D). The BTH containing exudate from MgCl_2_-infiltrated wt plants (red bars) conferred SAR only on wt plants (*P* < 0.005), whereas BTH containing exudate from *avrRpt2*-infiltrated wt plants (yellow bars) conferred SAR on both wt and *acbp* plants (*P* < 0.005). Notably, BTH slightly improved the SAR induced by *avrRpt2*-infiltrated wt exudate only on wt plants (*P* < 0.01, compare pink and yellow bars for each genotype infiltrated with Ex-Col-0). In comparison, the BTH containing exudate from MgCl_2_- or *avrRpt2-*infiltrated *acbp* plants was unable to confer SAR on either Col-0 or *acbp* plants. This suggested that the proper induction of SAR required a factor that was present in pathogen infected Col-0 exudates but absent in exudates from pathogen infected *acbp* plants. These data reconfirm that *acbp* mutants are defective in the generation of the mobile signal but not its perception and that SA alone is not sufficient for the induction of SAR.

**Figure 7:**
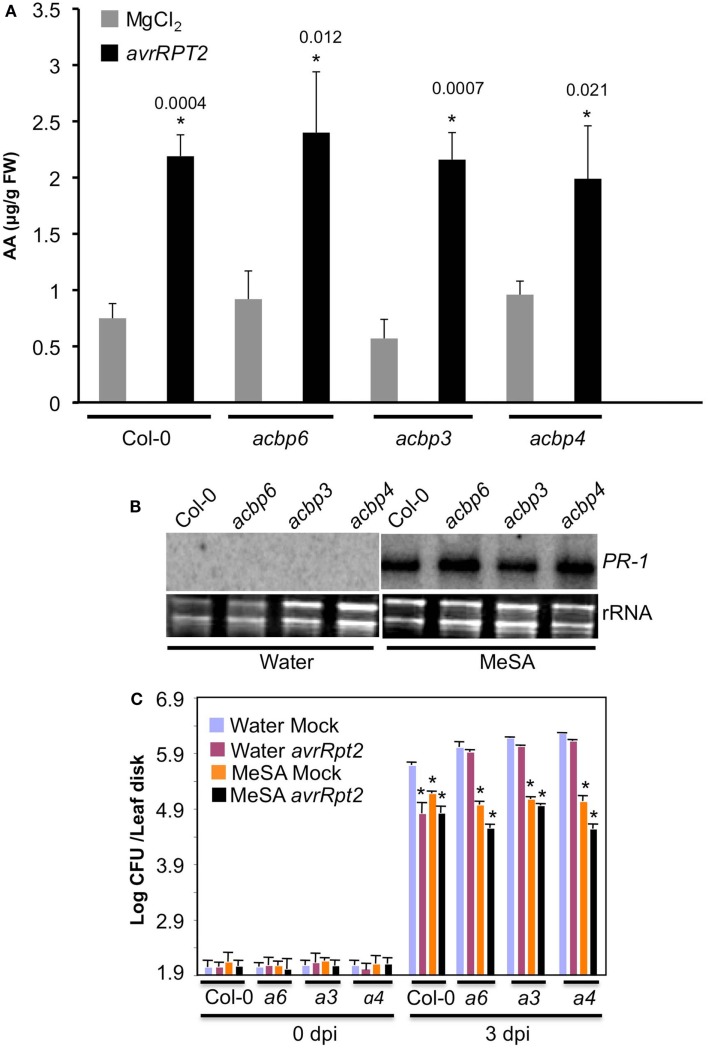
**The *acbp* mutants are responsive to MeSA and accumulate normal levels of AA**. **(A)** AA levels in mock (MgCl_2_, gray bars) and *avrRpt2* (black bars) inoculated wild-type (Col-0) and *acbp* mutants. Error bars indicate SD (*n* = 3). Statistical significance was calculated using Student’s *t*-test. Asterisks denote significant differences from mock-inoculated plants of corresponding genotype. Numbers above black bars indicate *P* values. The experiment was repeated twice with similar results. **(B)** RNA gel blot showing transcript levels of *PR-1* gene in plants treated with water or MeSA for 48 h. Ethidium bromide staining of total RNA was used as the loading control. The experiment was repeated twice with similar results. **(C)** SAR response in Col-0 and *acbp6* (*a6*), *acbp3* (*a3*), *acbp4* (*a4*) plants, pretreated with water (blue and pink bars) or 100 μM MeSA (orange and black bars) prior to infiltration with MgCl_2_ (mock, blue, and orange bars) or *P. syringae* expressing *avrRpt2* (pink and black bars). Bacterial growth presented as the LOG of colony forming units (CFU) per leaf disk, was monitored at 0 and 3 dpi. Error bars indicate SD (*n* = 4). Statistical significance was calculated using Student’s *t*-test (*P* < 0.005).

Recently, a dicarboxylic acid, azelaic acid (AA) was shown to confer SAR by priming biosynthesis of SA (Jung et al., 2009). To test if reduced accumulation of SA in *acbp* plants was due to compromised AA biosynthesis/accumulation, we monitored AA levels in mock- and pathogen inoculated wt and *acbp* petiole exudates (Figure 7A). The wt-like basal and pathogen-induced AA levels in *acbp* plants suggest that these are not altered in the biosynthesis and/or accumulation of AA. We next tested if *acbp* mutants were capable of converting the biologically inactive MeSA to SA, since conversion of the methylated ester of SA (MeSA) to SA is also critical for SAR (Seskar et al., 1998; Park et al., 2007). The wt and *acbp* plants were treated with MeSA for 48 h and evaluated for *PR-1* expression and SAR. The *acbp* mutants induced wt-like expression of *PR-1* in response to exogenous application of MeSA (Figure 7B), suggesting that these plants are capable of converting MeSA to SA. This was further supported by the fact that MeSA treated *acbp* mutants showed increased resistance against virulent pathogen (Figure 7C, *P* < 0.005). Together, these results suggest that compromised SA levels in pathogen inoculated *acbp* mutants were not due to defects in AA metabolism or the release of SA from the MeSA pool.

## Discussion

The *Arabidopsis* genome encodes six ACBPs, which localize to different cellular compartments. This study evaluated the defense related roles of *ACBP3*, *ACBP4*, and *ACBP6* gene products, which are well known to bind FA-CoA and/or various lipids (Xiao and Chye, 2009; Xiao et al., 2010), and are likely involved in their transport. We show that *ACBP3*, *ACBP4*, and *ACBP6* are required for basal resistance to fungal pathogens and, both basal (PTI) and *R*-mediated (ETI) resistance to bacterial pathogens. Notably, the *acbp3*, *acbp4*, and *acbp6* mutants are also defective in the induction of SAR. It is possible that the impaired PTI and/or ETI in these plants affect their abilities to induce SAR. However, it is also likely that the impaired SAR is associated with the defective cuticles in these mutants. This is supported by the fact that defective cuticular phenotype is associated with compromised SAR and bacterial resistance, and in some cases increased susceptibility to fungi as well as. However, unlike other cuticle defective mutants [like *acp* *4*; and *glabara* (*gl*) *1*; Xia et al., 2009, 2010], *acbp* plants are able to perceive the SAR mobile signal from wt plants, but cannot generate it themselves. It is possible that the extent of cuticular damage influences the plant’s ability to perceive the SAR signal(s) in the distal tissue since the cuticular defects of *acp4* and *gl1* plants are far more severe than those of the *acbp* mutants. Additionally, some of the specific signal(s) required for generation and/or perception of SAR may also contribute to cuticle development, therefore even though the *acp4* and *acbp* mutants are defective in different aspects of SAR they each have defective cuticles. The fact that cuticle contains glycerol (Molina et al., 2006; Pollard et al., 2008), which serves as a precursor for the SAR inducer G3P (Chanda et al., 2011), supports such a notion. Another possibility is that generation of the SAR signal(s) requires ACBP-mediated FA/lipid flux. Although this scenario does not explain the SAR defect in *acbp6* plants, which showed normal FA/lipid profile, we cannot discount the possibility that changes in the flux of some metabolite(s) were undetectable in our FA/lipid profiling procedures.

Interestingly, similar to *acbp* mutants, the cuticle defective *fad7 gl1* (but not *acp4*) mutant is also compromised in pathogen-induced SA levels, even though *fad7 gl1* is competent in SAR signal generation. It is possible that the defect in SA biosynthesis contributes to the enhanced susceptibility of the *acbp* mutants to virulent bacteria and fungal pathogens. However, exogenous application of the SA analog, BTH, together with avirulent pathogen was unable to restore SAR in *acbp* plants. This is not due to defects in AA biosynthesis or inability to convert MeSA to SA, because the *acbp* mutant plants show wt-like responsiveness to MeSA and contain wt-like AA levels. However, the whole plant-treatment experiments done here cannot discount the possibility that *acbp* mutants might be defective in MeSA generation in the primary infected leaves. Besides AA and MeSA, G3P, DA, and an amino acid derivative pipecolic acid (Pip), also regulate SAR, where DA and Pip trigger the accumulation of SA (Chaturvedi et al., 2012; Dempsey and Klessig, 2012; Zeier, J., personal communications). The interrelationships between these various SAR signals and their relationship to SA remain unclear. It will be useful to determine the levels of various SAR inducers and test their SAR inducing capabilities in cuticle defective plants.

Interestingly, in contrast to *acbp*, and *gl1* mutants, the damaged cuticle in *lacs2*, *lcr*, or fungal cutinase-overexpressing transgenic plants confers increased resistance to the necrotrophic pathogen *B. cinerea* and *Sclerotinia* (Bessire et al., 2007; Tang et al., 2007). Likewise, reduced surface hydrophobicity of mutants defective in abaxial epicuticular wax biosynthesis confers increased resistance to rust and anthracnose pathogens because the spores of these pathogens are unable to differentiate on these mutants (Uppalapati et al., 2012). On the other hand, loss of cuticle in the *gpat4 gpat8* double mutant is associated with enhanced susceptibility to *Alternaria brassicicola* (Li et al., 2007). These results suggest that cuticle permeability is not always associated with increased resistance (Voisin et al., 2009), and structural and/or physiological properties of cuticle might play diverse role(s) in different host-pathogen interactions.

Impaired lipid levels in *acbp3* and *acbp4* plants suggest that these ACBPs are required for maintaining normal lipid levels. Mutations in both *ACBP3* and *ACBP4* result in reduced MGDG, PG, and PC levels. In addition, *acbp3* plants are significantly reduced in MGDG and DGDG derived from the eukaryotic pathway (containing 18:3 species). This suggests that ACBP3 may be involved in the transport of DAG, the precursor of DGDG synthesis, from the ER (site of lipid biosynthesis via eukaryotic pathway) to the plastids (site of lipid biosynthesis via prokaryotic pathway). Both *acbp3* and *acbp4* plants are also affected in 16:3 FA containing MGDG and DGDG lipids, suggesting that these mutations also affect the prokaryotic pathway. Notably, the lipid profile and/or total lipid levels did not correlate with cuticular defects, since the cuticle defective *acbp6* plants show wt-like lipid profiles. The fact that mutations reducing total and plastidal lipids MGDG and DGDG do not always affect cuticle formation (Xia et al., 2010; Chanda et al., 2011), suggests that the flux of lipids or lipid precursors, rather than their levels, might be important for cuticle development. This is also consistent with the fact that the defect in cuticle development is more severe in *acbp3* mutant plants, which shows highest reduction in plastidal lipids species derived from the eukaryotic pathway. Notably, reduced lipid levels in *acbp3* and *acbp4* mutants are not associated with reduced FA pools. A likely explanation is that *acbp* mutants hyper-produce FA species to compensate for their deficiencies. This assumption is supported by the fact that several other cuticle defective mutants (Kurdyukov et al., 2006; Voisin et al., 2009) are hyperactive in the synthesis of cuticle components.

The compromised basal resistance to bacterial pathogen in *acbp3* mutant plants is consistent with a recent report showing increased resistance to virulent *P. syringae* in plants overexpressing *ACBP3* (Xiao and Chye, 2011). The *ACBP3* overexpressing plants accumulated higher basal levels of SA and showed constitutive cell death and *PR* expression. Consistent with these results, *acbp3* mutant plants are unable to accumulate wt-like levels of SA in response to pathogen infection. This in turn is consistent with the reduced expression of pathogen-responsive *PR-1* in these plants (data not shown). The impaired cuticle of *acbp3* mutants is unlikely to be associated with their reduced SA accumulation because, the *acp4* mutant, which also contains defective cuticle, is able to accumulate wt-like levels of SA in response to pathogen infection (Xia et al., 2009). Inoculation with virulent pathogen has been suggested to induce degradation and/or relocalization of ACBP3-GFP (Xiao and Chye, 2011), suggesting that ACBP3 might serve as one of the potential pathogen targets, which upon degradation leads to enhanced pathogen growth. Interestingly, *ACBP* has also been show to participate in senescence; the *acbp3* mutant shows delayed senescence and *ACBP3* overexpression accelerated starvation-induced leaf senescence (Xiao et al., 2010). Notably, the accelerated senescence in *ACBP3* overexpressing plants was dependent on the SA pathway, which is known to contribute to senescence (reviewed in Vincente and Plasencia, 2011). In this regard, it is interesting to note that delayed senescence is associated with the cuticular defect in the *lacerata* mutant (Wellesen et al., 2001). Clearly, more work is required to clarify the relationship between the phenotypes related to cuticle, senescence, and transport of FA/lipids in the *acbp* mutants, and the precise roles of ACBPs in these physiological processes.

## Materials and Methods

### Plant growth conditions

Plants were grown in MTPS 144 Conviron (Winnipeg, MB, Canada) walk-in chambers at 22°C, 65% relative humidity, and 14 h photoperiod. These chambers were equipped with cool white fluorescent bulbs (Sylvania, FO96/841/XP/ECO). The photon flux density (PFD) of the day period was 106.9 μmol m^−2^ s^−1^ (measured using a digital light meter, Phytotronic, Inc, MO, USA). Plants were grown on autoclaved Pro-Mix soil (Premier Horticulture, Inc., PA, USA). Soil was fertilized once using Scotts Peter’s 20:10:20 peat lite special general fertilizer that contained 8.1% ammoniacal nitrogen and 11.9% nitrate nitrogen (Scottspro.com). Plants were irrigated using deionized or tap water. The *acbp3*, *acbp4*, and *acbp6* T-DNA mutants were identified from the SALK-012290, SALK-040164, and SALK-104339 lines, respectively. The genotypes were determined by PCR. The SALK lines used here have been used in several earlier studies (Chen et al., 2008; Xiao et al., 2008, 2010).

### RNA extraction, northern, and PCR analyses

Small-scale extraction of RNA from one or two leaves was performed in the TRIzol reagent (Invitrogen, Gaithersburg, MD, USA) following the manufacturer’s instructions. RNA gel blot analysis and synthesis of random primed probes was carried out as described before (Kachroo et al., 2005). RNA quality and concentration were determined by gel electrophoresis and determination of A260. Reverse transcription (RT) and first strand cDNA synthesis was carried out using Superscript II (Invitrogen). Two to three independent RNA preparations were used for RT-PCR and each of these were analyzed at least twice by RT-PCR. The RT-PCR using gene-specific primers was carried out for 35 cycles in order to determine absolute levels of transcripts.

### SA quantification

SA and SAG were extracted and measured from ∼ 0.3 g of fresh weight leaf tissue, as described before (Chandra-Shekara et al., 2006). For SA measurements plants were inoculated with 10^5^/ml bacteria and samples were harvested 48 h post inoculation. Data presented are a mean of three biological repeats.

### AA, FA, lipid, and glucose analyses

For AA estimations, petiole exudates were extracted using a solution containing glacial acetic acid, methanol, chloroform, and potassium chloride (0.9%; 1:4:8:8 V/V) and 17:0 as the internal standard. The lower phase was dried under compressed nitrogen and samples were derivatized with MTBSTFA containing 1% TBDMCS, suspended in acetonitrile and analyzed by gas chromatography (GC) on a Varian FAME 0.25 mm × 50 mm column equipped with mass spectrometer (MS; Hewlett Packard).

Extraction of total FAs was carried out by placing leaf tissue in 2 ml of 3% H_2_SO_4_ in methanol. After 30 min incubation at 80°C, 1 ml of hexane with 0.001% butylated hydroxytoluene (BHT) was added. The hexane phase was then transferred to vials for GC analysis. One-microliter samples were analyzed by GC on a Varian FAME 0.25 mm × 50 mm column and quantified with flame ionization detection. FAs were identified based on their retention time relative to known FA standards. For quantification of FAs, leaves (50 mg) were extracted together with an internal standard 19:0 and the FA levels were calculated based on the detected peak areas corresponding to the FA retention time relative to the areas of the internal standard. FA analysis is representative of at least four independent repeats.

For lipid extraction, six to eight leaves were incubated at 75°C in isopropanol containing 0.001% BHT for ∼15 min. To this, 1.5 ml chloroform and 0.6 ml water was added and the samples were agitated at room temperature for 1 h. The lipids were re-extracted in chloroform: methanol (2:1) until the leaves were bleached. The aqueous content was removed by partitioning with 1 M KCl and water. The lipid extract was dried under a gentle stream of nitrogen gas and re-dissolved in 0.5 ml of chloroform. Lipid analysis and acyl group identification was carried out with five biological replicates using the automated electrospray ionization-tandem mass spectrometry facility at Kansas Lipidomics Research Center (Welti et al., 2002).

Glucose was quantified as described before (Chanda et al., 2008).

### SA, MeSA, and BTH treatments

SA and BTH were dissolved in water and the pH of SA solution was adjusted to 6.5 with KOH. SA and BTH treatments were carried out by spraying 500 μM, or 100 μM solutions, respectively, until runoff. MeSA (Sigma-Aldrich, Inc.) was dissolved in 100 μl of methanol and diluted in water to 100 μM working concentration.

### Pathogen infections

Inoculations with bacterial pathogen *P. syringae* were conducted as described before (Kachroo et al., 2005). The bacterial cultures were grown overnight in King’s B medium (King et al., 1954) containing 50 μg/ml of rifampicin and/or kanamycin. The cells were washed and suspended in 10 mM MgCl_2_. The bacterial suspension was injected into the abaxial surface of the leaf using needle-less syringe. Three disks from the inoculated leaves were collected and homogenized in 10 mM MgCl_2_. The extract was diluted and appropriate dilutions were plated on King’s B medium. For analysis of SAR, the primary leaves were inoculated with MgCl_2_ or the avr bacteria (10^7^ CFU ml^−1^) and 48 h later the distal leaves were inoculated with vir bacteria (10^5^ CFU ml^−1^). Unless noted otherwise, samples from the distal leaves were harvested at 3 dpi and monitored for growth of virulent bacteria.

*Colletotrichum higginsianum* Sacc. (IMI 349063) and *B. cinerea* were maintained on potato dextrose agar (PDA; Difco) and V8 medium (Kent et al., 2008), respectively. Four-week-old *Arabidopsis* plants were used for both spray and spot inoculations. Fungal spores were harvested by scrapping the surface of cultures maintained on PDA or V8 plates, washed three to four times with sterile water, quantified using a hemocytometer, and suspended at concentrations of 10^4^ to 10^6^ spores/ml. For spot inoculations, 10 μl of spore suspension was used to inoculate *Arabidopsis* leaves. After inoculations, the plants were transferred to a PGV36 Conviron walk-in chamber and covered with a plastic dome to maintain high humidity. Disease symptoms were scored between 4 and 11 dpi. A digital Vernier caliper was used to measure lesion size in spot-inoculated leaves. Each experiment was repeated at least twice and each included 30–50 individual plants. Statistical significance was determined using Student’s *t*-test.

### Collection of petiole exudate

Petiole exudate was collected as described earlier (Maldonado et al., 2002). In brief, plants were induced for SAR by inoculation with *P. syringae* containing *avrRpt2* (10^6^ CFU ml^−1^). Twelve 24 h later, petioles were excised, surface sterilized in 50% ethanol, 0.0006% bleach, rinsed in sterile 1 mM EDTA and submerged in ∼1.9 ml of 1 mM EDTA and 100 μg ml^−1^ ampicillin. Exudates were collected over 48 h and infiltrated into healthy plants. Infiltrated leaves were harvested after 2 days for *PR-1* gene expression studies. For SAR studies, vir pathogen was inoculated in the distal leaves 2 days after infiltration of exudate.

### Toluidine blue staining

Leaf samples were taken from 4-week-old plants grown on soil and stained with toluidine blue staining was carried out as described earlier (Tanaka et al., 2004). Each genotype was tested in five to six independent experiments with a total of 30–50 leaves stained.

### Microscopy, chlorophyll leaching, and water loss

For SEM analysis both abaxial and adaxial surface of the leaf samples was mounted on sample holder with 12 mm conductive carbon tabs (Ted Pella, Inc.), sputter-coated with gold-palladium and observed on a Hitachi S-3200 SEM, with and without backscatter detector at 5 and 20 kV. Two to three leaves were observed per genotype.

For TEM analysis leaves were fixed in paraformaldehyde and embedded in epon-araldite. Leaves were sectioned on a Reichert–Jung Ultracut E microtome with a Diatome diamond knife and observed under a Philips Tecnai Biotwin 12 TEM. Three to four sections were analyzed per genotype.

For chlorophyll leaching assays, 100 mg of leaves were weighed and gently agitated, in dark, at room temperature in tubes containing 80% ethanol. Absorbance of each sample was measured at 664 and 647 and micromolar concentration of total chlorophyll per gram of fresh weight was calculated using the formula: total micromoles chlorophyll = 7.93 (A_664_) + 19.3 (A_647_).

For water loss in response to drought treatment, 4-week-old plants were left unwatered until the soil dried completely. The leaf weight was measured from ∼50 leaves.

### Analysis of wax and cutin components

For analysis of the wax component, 500 mg of 4-week-old leaves were immersed in 10 ml of chloroform for 10 s. The leaves were rinsed once more with 10 ml of chloroform. An internal standard (100 μg of *n*-tetracosane) was added and the sample volume was evaporated under a gentle steam of nitrogen. The samples were dried under a stream of nitrogen gas and methylated with diazomethane, dried again, and derivatizated with 100 μl of acetic anhydride in 100 μl of pyridine and the sealed tubes were incubated for 60 min at 60°C. The samples were again dried under a stream of nitrogen and dissolved in 1 ml of acetonitrile. Samples (1 μl) were injected into an HP-5 column (injection temperature 250°C) of GC equipped with flame ionization detector (temperature 300°C). The same samples were also run on an HP-5 column (30 mm × 0.32 mm × 0.25 mm film thickness) on a GC equipped with MS. Various components were identified based on their retention time as compared to standards and by MS analysis. Quantification was based on flame ionization detector peak areas as compared to the peak areas of the internal standard tetracosane added prior to derivatization.

Cutin monomer composition and content were determined using sodium methoxide-catalyzed transmethylation method followed by acetylation of the hydroxyl groups with acetic anhydride and GC-MS slightly modified from previously described (Bonaventure et al., 2004; Molina et al., 2006). After methanolysis, the methylene dichloride extract of cutin monomers were washed with 0.9% potassium chloride instead of 0.5 M sodium chloride. For GC-MS analysis, the FAME capillary column used was as described in wax analysis with helium carrier gas at 1 ml min^−1^. The MS was run in scan mode over 35–450 amu (electron impact ionization).

## Conflict of Interest Statement

The authors declare that the research was conducted in the absence of any commercial or financial relationships that could be construed as a potential conflict of interest.
